# Role of Synbiotics Containing d-Allulose in the Alteration of Body Fat and Hepatic Lipids in Diet-Induced Obese Mice

**DOI:** 10.3390/nu10111797

**Published:** 2018-11-19

**Authors:** Bo-Ra Choi, Eun-Young Kwon, Hye-Jin Kim, Myung-Sook Choi

**Affiliations:** 1 Department of Food Science and Nutrition, Kyungpook National University, Daegu 41566, Korea; borachoi15@naver.com (B.-R.C.); savage20@naver.com (E.-Y.K.); 2 Center for Food and Nutritional Genomics Research, Kyungpook National University, Daegu 41566, Korea; 3 Food R&D, CJ Cheiljedang Corp., 55, Gwanggyo-ro 42beon-gil, Yeongtong-gu, Suwon-si, Gyeonggi-do 16495, Korea; pinkponys@naver.com

**Keywords:** d-allulose, prebiotics, obesity, body fat reduction

## Abstract

The effects of allulose and two probiotic species on diet-induced obese (DIO) mice were investigated. *Lactobacillus sakei* LS03 (10^9^ cfu/day) and *Leuconostoc kimchii* GJ2 (10^9^ cfu/day) were used as probiotics, and allulose (AL) as a prebiotic. The synergistic effect of prebiotics and probiotics in improving obesity was evaluated. Orally fed *Lactobacillus sakei* LS03 (LS) or *Leuconostoc kimchii* GJ2 (GJ), significantly decreased hepatic triglyceride (TG) and fatty acid (FA) compared to the high-fat diet (HFD) control. AL markedly decreased visceral adiposity and pro-inflammatory adipokines (leptin and resistin) and cytokines (IL-6 and IL-1β) as well as hepatic TG and FA. In addition, AL exerted synergic effects with probiotics (LS and/or GJ) on the reduction of visceral white adipose tissue (WAT), associated with a decreased leptin: adiponectin ratio. There was no significant differences between the AL-SL and AL group, allulose and GJ combination (AL-GJ) was more effective than allulose in improving dyslipidemia, and decreasing WAT weight and hepatic FA, suggesting allulose may act as a favorable prebiotic for GJ supplement than LS. Combination of allulose with LS and GJ supplementation (AL-LSGJ) was the most effective for improving obesity related complications among the synbiotics groups containing allulose. In conclusion, this study demonstrated that the synbiotic mixture with allulose was more effective in suppressing diet-induced obese (DIO) and its complications via the regulation of lipid metabolism, than the probiotics or allulose alone, suggesting allulose may act as a prebiotic for the two probiotics tested in the study. This new synbiotic mixture with allulose may help ameliorate the deleterious effects of diet-induced obesity and contribute to the growth of the food industry.

## 1. Introduction

Prevalence of obesity is considered one of the most important health problems worldwide. Obesity is characterized by an abnormal excess of white adipose tissue, which is a major risk factor for developing diabetes, dyslipidemia, and liver steatosis [1]. Combinations of probiotics and prebiotics, defined as synbiotics, are consumed in diverse forms including yogurt, cheese, and several types of fermented food [2]. Reportedly, synbiotics may enhance the prevention and treatment of dyslipidemia, nonalcoholic fatty liver disease, and obesity [3,4,5]. Therefore, the development of new synbiotics may contribute to the growth of the food industry and enhance the nutrition and health of consumers [6].

In this study, *Lactobacillus sakei* LS03 and *Leuconostoc kimchii* GJ2 were used as probiotics. These probiotics were isolated from kimchi, a traditional Korean fermented vegetable. The genera *Lactobacillus* and *Leuconostoc,* used as starter cultures in kimchi fermentation, are the dominant lactic acid bacteria of kimchi [7]. Previous studies have reported that *Lactobacillus sakei* OK67 may contribute to lowering epididymal fat accumulation and pro-inflammatory factors such as tumor necrosis factor-alhpa (TNF-α), interleukin-6 (IL-6) and interleukin-1 beta (IL-1β) [8]. Jo et al. reported that *Leuconostoc kimchii* GJ2 may improve cardiovascular diseases and hypercholesterolemia by reducing serum cholesterol, triglyceride and low-density lipoprotein cholesterol levels [9]. We tested the effects of d-allulose as a prebiotic. d-allulose, a low-calorie (0.2 kcal/g) functional sweetener, has been recently reported as being beneficial for the prevention of obesity due to its anti-hyperlipidemic effect, brought on by the altering of lipid-regulating enzyme activities and gene expressions levels [10,11]. Moreover, Kimoto-Nira et al. has reported that d-allulose may have a beneficial effect on the growth and activity of probiotics in vitro [12]. Thus, further investigative studies on the potential use of d-allulose as a prebiotic in preventing and ameliorating obesity and its complications are felt to be necessary.

This study was undertaken to evaluate whether allulose may act synergistically with two probiotic species such as *Lactobacillus sakei* LS03 and *Leuconostoc kimchi* GJ2 in reducing obesity. We measured body weight and white adipose tissue weight, and analyzed lipid profiles in the plasma, hepatic enzyme activities related to lipid metabolism, as well as gene expression in the liver and epididymal white adipose tissue.

## 2. Materials and Methods

### 2.1. Animals and Diets

Five weeks old male C57BL/6J mice (*n* = 70) were purchased from the Jackson Laboratory (Bar Harbor, ME, USA), and bred in another institution (KPC, Gwangju, Gyeonggi Province, Republic of Korea). d-allulose and the probiotics were supplied by CJ CheilJedang Corp (Seoul, Korea). The mice were maintained in a room with controlled temperature (20–25 °C) under 12 h dark/light alternating conditions. The mice were fed commercial chow diet pellets during the 1 week adaptation period. Next, the mice were fed a normal diet (ND; 5% fat (corn oil), *w*/*w*) for 16 weeks and a high-fat diet (HFD; 20% fat, 1% cholesterol, *w*/*w*) for 4 weeks to induce obesity. HFD contained 40 kcal% fat, 17 kcal% protein, and 43 kcal% carbohydrate with the fat sources consisting of lard (85% (*w*/*w*) of total fat) and corn oil (15% (*w*/*w*) of total fat). After induction of obesity, the mice fed a HFD were randomly divided into seven groups and fed HFD (*n* = 10), HFD with *Lactobacillus sakei* LS03 at 10^9^ cfu/day (LS; *n* = 10), HFD with *Leuconostoc kimchi* GJ2 at 10^9^ cfu/day (GJ; *n* = 10), HFD with 3% allulose at 3% allulose substituted for sucrose in HFD, *w*/*w* (allulose (AL); *n* = 10), HFD with allulose and *Lactobacillus sakei* LS03 at 10^9^ cfu/day (AL-LS; *n* = 10), HFD with allulose and *Leuconostoc kimchi* GJ2 10^9^ cfu/day (AL-GJ; *n* = 10), and HFD with allulose, *Lactobacillus sakei* LS03 at 10^9^ cfu/day and *Leuconostoc kimchi* GJ2 at 10^9^ cfu/day (AL-LSGJ; *n* = 10) for 12 weeks (Table 1). The mice had free access to water and food, except probiotics, during the entire experimental period. Diets were supplied in pellet form and fresh probiotics were orally administered daily during the experimental period. Probiotics were dissolved in 250 μL of phosphate buffered saline (PBS) and the amounts of probiotics given were 10^9^ cfu/day. Just PBS, without probiotics, was orally administered to the control, HFD and AL groups. After 12 weeks, all mice were anesthetized with diethyl ether and sacrificed for 16 h fast. Blood was taken from the inferior vena cava for plasma analysis. The liver and adipose tissue were removed, rinsed with physiological saline, weighed and immediately frozen in liquid nitrogen, and stored at −80 °C until analysis. The animal study protocols were approved by the Ethics Committee at KPC (approval no. P150067).

### 2.2. Plasma Biomarkers

Plasma lipid concentration was determined using commercially available kits. Plasma triglyceride (TG), total-cholesterol (TC), high-density lipoprotein cholesterol (HDL-C), glutamic oxaloacetic transaminase (GOT), and glutamic pyruvic transaminase (GPT) levels were determined using Asan enzymatic kits (Asan, Seoul, Korea). Plasma apolipoprotein AI (ApoA-I) and apolipoprotein B (ApoB) were measured using enzymatic kits (Eiken, Japan). Plasma free fatty acid (FFA) was measured using Nittobo enzymatic kit (Nittobo Medical Co., Tokyo, Japan). Plasma adipokines (leptin, resistin, and adiponectin) and cytokines (TNF-α, IL-6 and IL-1β) were determined via a multiplex detection kit from Bio-Rad (Hercules, CA, USA). Data analyses were performed using Bio-Plex Manager software version 4.1.1 (Bio-Rad, Hercules, CA, USA).

### 2.3. Hepatic Lipid Contents

Hepatic lipid was extracted [13], and dried lipid residues were dissolved in 1 mL of ethanol for triglyceride, cholesterol, and fatty acid assays. A solution of Triton X-100 and sodium cholate in distilled water was added to 200 μL of a dissolved lipid solution for the purpose of emulsification. Triglyceride, cholesterol, and fatty acid contents were analyzed using the same enzymatic kit used for the plasma analysis.

### 2.4. Hepatic Lipid-Regulating Enzymes Activities

Hepatic cytosolic and mitochondrial fractions were prepared and analyzed according to the method developed by Hulcher and Oleson [14]. Protein concentration in the enzyme sources was determined using the Bradford method. Glucose-6-phosphate dehydrogenase (G6PD) [15] and malic enzyme (ME) [16] activities were determined using the previously described method. Carnitine palmitoyl transferase (CPT) was determined using the method described by Markwell et al. [17]. Fatty acid β-oxidation was determined via Lazarow’s method [18].

### 2.5. Real-Time qPCR Analysis

Liver and epididymal white adipose tissue (WAT) was prepared from each group of mice. Total RNA, extracted using TRIzol reagent (Invitrogen, Grand Island, NY, USA), was used to synthesize cDNA via the QuantiTect Reverse Transcription kit (QIAGEN Gmbh, Hilden, Germany). RNA expression was quantified by way of a quantitative real-time polymerase chain reaction (PCR) using the QuantiTect SYBR Green PCR kit (QIAGEN Gmblh, Hilden, Germany). Primers were designed to detect fatty acid synthase (FAS), acetyl-CoA carboxylase 1 (ACC1), peroxisome proliferator-activated receptor alpha (PPARα), carnitine palmitoyltransferase 1-alpha and 2 (CPT1α and CPT2), peroxisome proliferator-activated receptor gamma coactivator 1-alpha (PGC1α), sirtuin 1 (SIRT1), and glyceraldehyde-3-phosphate dehydrogenase (GAPDH) (Table 2). The amplification was performed as follows: 10 min at 90 °C, 15 s at 95 °C, and 60 s at 60 °C, for a total of 35 cycles. The Ct data were normalized using GAPDH, and the relative gene expression level was calculated with the 2 ^∆∆^Ct method.

### 2.6. Histopathological Analysis

Liver samples from each mouse were removed and fixed in 10% (*v*/*v*) formalin/PBS buffer solution. Fixed samples were embedded in paraffin in preparation for staining with hematoxylin and eosin. Stained slices were viewed under an optical microscope (Nikon, Tokyo, Japan) set at 200 × magnification.

### 2.7. Statistical Analysis

All data were presented as mean ± standard error of the mean (SEM) and were analyzed using SPSS (SPSS Inc. Chicago, IL, USA). Statistical differences between the ND group and the HFD group were determined using Student’s *t*-test. One-way analysis of variance (one-way ANOVA) was used to evaluate statistical significance between groups fed the high-fat diet (HFD, LS, GJ, AL, AL-LS, AL-GJ, and AL-LSGJ). The statistical significance between group means was determined via Duncan’s multiple-range test, multiple comparison procedure at *p* < 0.05.

## 3. Results

### 3.1. Body Weights and Food Efficiency Ratio

**Figure 1:**
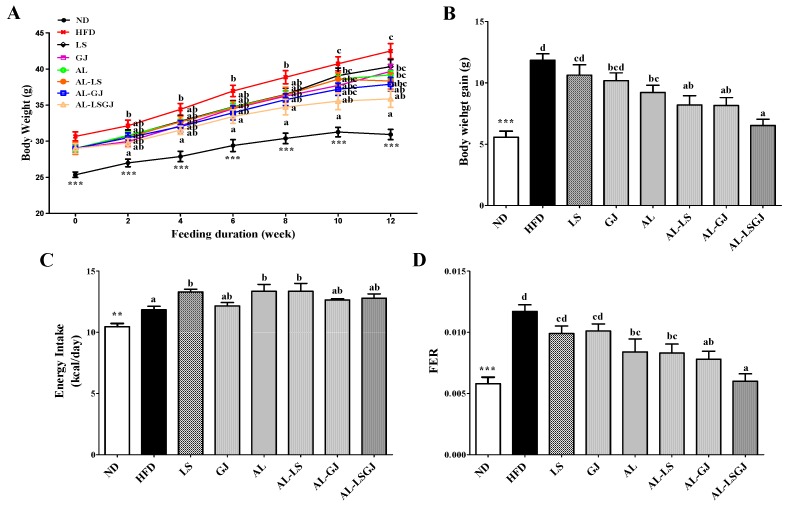
Effect of synbiotic supplementation for 12 weeks on body weight, measured weekly. (**A**) body weight, (**B**) body weight gain, (**C**) energy intake and (**D**) food deficiency ratio (FER) in diet-induced obese mice. Data represented as mean ± standard error of the mean (S.E.); Significant differences ND versus HFD are indicated; ** *p* < 0.01, *** *p* < 0.001; ^abcd^ Means with different superscript letters are significantly different among the groups (*p* < 0.05); ND: normal diet (5% fat, *w*/*w*); HFD: high fat diet (20% fat, 1% cholesterol, *w*/*w*); LS: HFD + *Lactobacillus sakei* LS03 10^9^ cfu/day; GJ: HFD + *Leuconostoc kimchi* GJ2 10^9^ cfu/day; AL: HFD + 3% allulose; AL-LS: AL + *Lactobacillus sakei* LS03 10^9^ cfu/day; AL-GJ: AL + *Leuconostoc kimchi* GJ2 10^9^ cfu/day; AL-LSGJ: AL + *Lactobacillus sakei* LS03 + *L. Leuconostoc kimchi* GJ2 10^9^ cfu/each/day; FER: food efficiency ratio = body weight gain/energy intake per day.

Supplementation of HFD significantly increased body weight (BW) and decreased food efficiency ratio (FER) throughout the experiment compared to the ND (Figure 1). Probiotic LS- and GJ-supplemented groups did not significantly affect BW gains and FER. AL significantly decreased the BW gain, despite the increase in energy intake. These led to a decreased FER in the AL group compared with the HFD group. Allulose with probiotic LS (AL-LS) significantly decreased the BW from 12th week, and allulose with probiotic GJ (AL-GJ) markedly decreased the BW from 6th week compared to HFD (Figure 1A). AL-LS and AL-GJ also exhibited a significant decrease in BW gain and FER when compared to the HFD group. Additionally, synergies were observed between allulose and probiotics in BW gain and FER. AL-LS tend to decrease the BW gain, and AL-GJ also tend to decrease both the BW gain and FER compared to the AL group. Furthermore, the AL-LSGJ group, that was fed allulose with two species of probiotics, had the lowest BW gain and FER among HFD-fed groups (Figure 1B,D). AL-LSGJ significantly decreased the BW from the second week when compared to the HFD group (Figure 1A). Similar to the BW results, the BW gain and FER were significantly lower in the AL-LSGJ group compared to that in the HFD, probiotics (LS, GJ), and AL groups (Figure 1B,D).

### 3.2. Adipose Tissue Weights

The weight of liver and all white adipose tissue depots (epididymal, perirenal, mesenteric, subcutaneous, retroperitoneal, and interscapular) were significantly increased, while kidney weight was markedly decreased by the HFD when compared to the ND (Table 3). Probiotic LS- and GJ-supplemented groups significantly decreased only interscapular WAT compared with the HFD group. AL group vs. HFD group exhibited significant decrease in the weights of perirenal, mesenteric, interscapular and visceral WAT (including epididymal, perirenal, retroperitoneal and mesenteric WAT depots). AL-LS showed a significant decrease in perirenal, mesenteric, interscapular, visceral and total WAT when compared to the HFD group. The AL-GJ and AL-LSGJ groups showed a dramatic decrease in all WAT depots compared to the HFD group. Additionally, a synergic effect between allulose and probiotics was observed. The AL-GJ markedly decreased the weights of subcutaneous interscapular and total WAT, and the AL-LSGJ significantly decreased the weights of all WAT depots, except retroperitoneal WAT, when compared to the AL group.

Kidney weight of the AL group was significantly increased compared to that of the HFD group. Supplementation of AL-LS and AL-GJ significantly increased kidney weight compared to LS and GJ, respectively. However, the kidney weight in the allulose-based groups (AL, AL-LS, AL-GJ, and AL-LSGJ) was not increased than in the ND group (Table 3).

### 3.3. Plasma Lipid Profiles

Supplementation of HFD significantly increased the plasma TG, TC, FFA, HDL-C, nonHDL-C, Apo B, and Apo A-I:Apo B ratio compared to the ND group (Table 4). The supplementation of GJ, AL and AL-LS significantly decreased the levels of plasma TG, non HDL-C and AI compared to the HFD. Conversely, the HDL-C:TC ratio (HTR) in the GJ, AL, and AL-LS groups was significantly higher than that in the HFD group. LS also significantly decreased non-HDL-C and AI, while increased HTR compared to the HFD. AL-GJ supplementation led to a significantly lower concentration of plasma TG, TC, nonHDL-C and ApoB, and AI, whereas HTR and the Apo A-I:Apo B ratio were significantly increased by AL-GJ supplementation compared to the HFD group. Moreover, the AL-LSGJ group fed allulose-supplemented HFD with 2 species of probiotics significantly altered all plasma lipid parameters (TG, TC, FFA, HDL-C, nonHDL-C, HTR, AI, Apo A-I, Apo B, and the Apo A-I:Apo B ratio compared to the HFD group. Especially, TC level was significantly decreased by AL-LSGJ, while the Apo A-I level and Apo A-I:Apo B ratio were significantly higher in the AL-LSGJ group when compared with the LS, GJ, and AL groups. Accordingly, these markers were lowest in the AL-LSGJ group in entire HFD-fed groups (Table 4).

### 3.4. Plasma Adipokine and Cytokine Levels

**Figure 2:**
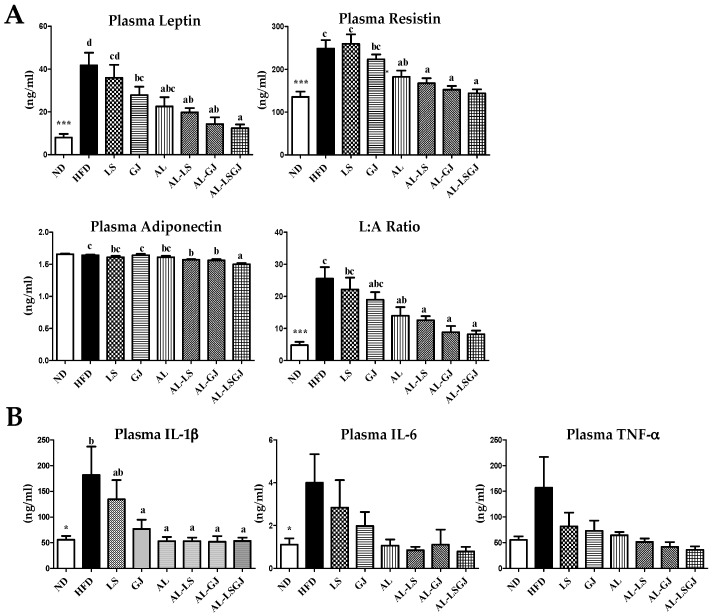
Effect of synbiotic supplementation for 12 weeks on plasma adipokine levels. (**A**) adipokine levels and (**B**) cytokine levels in diet-induced obese mice. Data are mean ± S.E.; Significant differences of ND versus HFD are indicated; * *p* < 0.05, *** *p* < 0.001; ^ab^^cd^ Means with different superscript letters are significantly different among the groups (*p* < 0.05); ND: normal diet (5% fat, *w*/*w*); HFD: high fat diet (20% fat, 1% cholesterol, *w*/*w*); LS: HFD + *Lactobacillus sakei* LS03 10^9^ cfu/day; GJ: HFD + *Leuconostoc kimchi* GJ2 10^9^ cfu/day; AL: HFD + 3% allulose; AL-LS: AL + *Lactobacillus sakei* LS03 10^9^ cfu/day; AL-GJ: AL + *Leuconostoc kimchi* GJ2 10^9^ cfu/day; AL-LSGJ: AL + *Lactobacillus sakei* LS03 + *L. Leuconostoc kimchi* GJ2 10^9^ cfu/each/day; IL-6: interleukin-6; IL-1β: interleukin-1 beta; TNF-α: tumor necrosis factor-alhpa.

The HFD group dramatically increased the level of plasma adipokines (leptin, resistin) and pro-inflammatory cytokines (IL-1β and IL-6) when compared to the ND group (Figure 2). LS did not significantly affect the plasma adipokine/cytokine profiles. GJ supplementation significantly decreased plasma leptin and IL-1β levels compard to the HFD group. The plasma leptin, resistin, leptin: adiponectin ratio (L:A ratio) and IL-1β were dramatically decreased in the AL, AL-LS, AL-GJ, and AL-LSGJ groups compared to those in the HFD group.

### 3.5. Hepatic Lipids Levels, Enzyme Activity, and mRNA Expression

**Figure 3:**
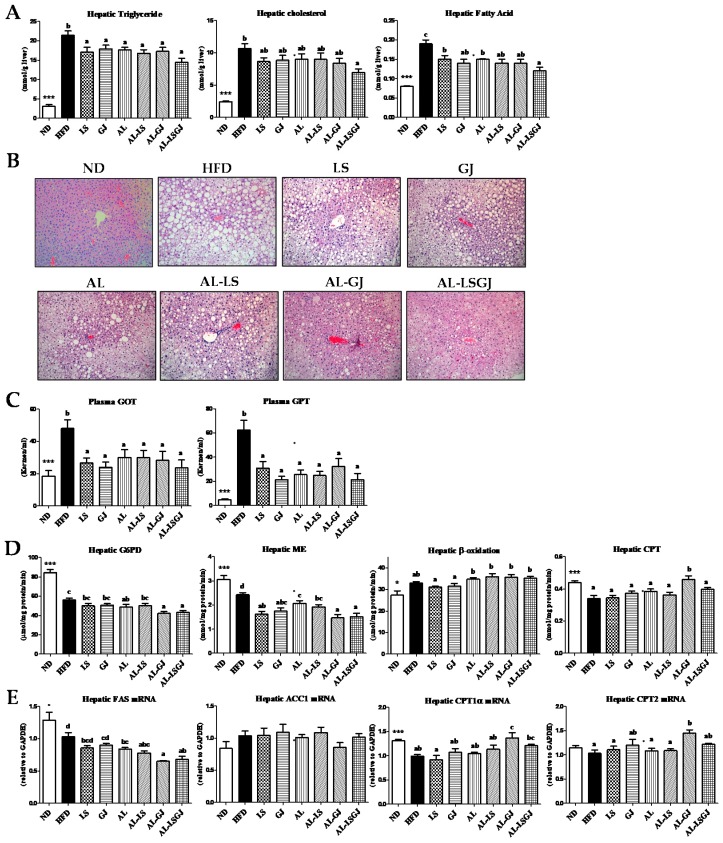
Effect of synbiotic supplementation for 12 weeks on hepatic morphology. (**A**) hepatic lipid levels, (**B**) hepatic morphology (X200), (**C**) hepatic lipotoxicity markers, (**D**) hepatic lipid regulating enzyme activities and (**E**) hepatic gene expressions in diet-induced obese mice. Data are mean ± S.E.; Significant differences ND versus HFD are indicated; * *p* < 0.05, *** *p* < 0.001; ^ab^^c^ Means with different superscript letters are significantly different among the groups (*p* < 0.05); ND: normal diet (5% fat, *w*/*w*); HFD: high fat diet (20% fat, 1% cholesterol, *w*/*w*); LS: HFD + *Lactobacillus sakei* LS03 10^9^ cfu/day; GJ: HFD + *Leuconostoc kimchi* GJ2 10^9^ cfu/day; AL: HFD + 3% allulose; AL-LS: AL + *Lactobacillus sakei* LS03 10^9^ cfu/day; AL-GJ: AL + *Leuconostoc kimchi* GJ2 10^9^ cfu/day; AL-LSGJ: AL + *Lactobacillus sakei* LS03 + *L. Leuconostoc kimchi* GJ2 10^9^ cfu/each/day; GOT: glutamic oxaloacetic transaminase; GPT: glutamic pyruvic transaminase; G6PD: Glucose-6-phosphate dehydrogenase; ME: malic enzyme; FAS: fatty acid synthase; ACC1: acetyl-CoA carboxylase 1; CPT: carnitine palmitoyltransferase.

The HFD group dramatically increased the levels of hepatic lipids (TC, TG, and FFA) and hepatic lipotoxicity markers (plasma GOT and GPT) when compared to the ND group (Figure 3A). The levels of hepatic TG and FFA were significantly lower in the allulose- and probiotics-fed groups (LS, GJ, AL, AL-LS, AL-GJ, and AL-LSGJ) compared to those in the HFD group (Figure 3A). Especially, AL-LSGJ supplementation markedly decreased hepatic TC as well as TG and FFA compared to the HFD, and hepatic FA level in the AL-LSGJ group was markedly lower than those in the LS, GJ, and AL groups. Similar to the hepatic lipid levels, hematoxylin and eosin (H&E) staining results indicated that hepatic lipid droplets numbers and sizes were decreased in AL-LSGJ-treated mice compared to those in other groups. In addition, levels of plasma GOT and GPT were also reduced in the allulose- and probiotics-fed groups than in the HFD group (Figure 3B,C).

The activities of hepatic enzymes related to FA synthesis (G6PD and ME) were significantly decreased by AL supplementation compared to HFD (Figure 3D). Moreover, the activity of ME in LS- and GJ-groups was significantly reduced compared to that of the HFD group. Also, G6PD activity was significantly lowered in both AL-GJ- and AL-LSGJ-groups than in the LS and GJ groups, respectively, and ME activity was significantly lowered in both AL-GJ- and AL-LSGJ-groups than in the AL group. Activity of β-oxidation was tended to be higher in the allulose-fed groups (AL, AL-LS, AL-GJ, and AL-LSGJ) compared to that in the HFD group. Hepatic CPT activity was significantly increased in the AL-GJ group only compared to those in the HFD group. The mRNA expression of *FAS* was significantly lower in the allulose treated groups (AL, AL-LS, AL-GJ, and AL-LSGJ) compared to that in the HFD group (Figure 3E). The AL-GJ group markedly reduced hepatic *FAS* mRNA expression compared to LS, GJ and AL groups. By contrast, mRNA expression of *CPT1α* and *CPT2* were significantly up-regulated by the AL-GJ treatment compared to the HFD group. Furthermore, mRNA expression of *CPT1α* was dramatically increased by AL-GJ treatment compared to the LS, GJ, and AL treatment.

### 3.6. mRNA Expression in Epididymal White Adipose Tissue

**Figure 4:**
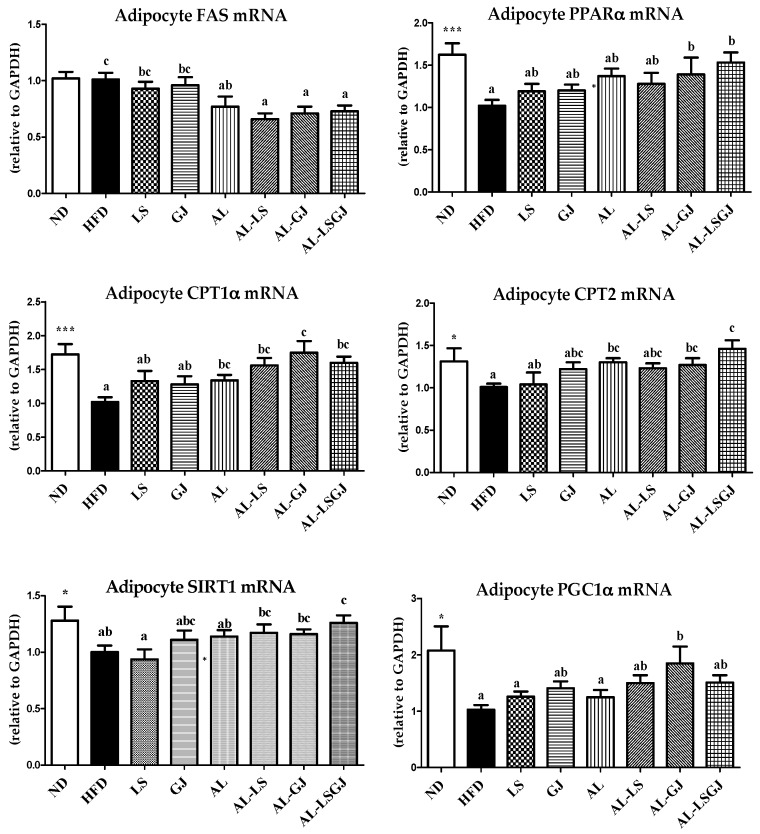
Effect of synbiotic supplementation for 12 weeks on epididymal adipose tissue gene expressions in diet-induced obese mice. Data are mean ± S.E.; Significant differences ND versus HFD are indicated; * *p* < 0.05, *** *p* < 0.001; ^abc^ Means with different superscript letters are significantly different among the groups (*p* < 0.05); ND: normal diet (5% fat, *w*/*w*); HFD: high fat diet (20% fat, 1% cholesterol, *w*/*w*); LS: HFD + *Lactobacillus sakei* LS03 10^9^ cfu/day; GJ: HFD + *Leuconostoc kimchi* GJ2 10^9^ cfu/day; AL: HFD + 3% allulose; AL-LS: AL + *Lactobacillus sakei* LS03 10^9^ cfu/day; AL-GJ: AL + *Leuconostoc kimchi* GJ2 10^9^ cfu/day; AL-LSGJ: AL + *Lactobacillus sakei* LS03 + *L. Leuconostoc kimchi* GJ2 10^9^ cfu/each/day; PPARα: peroxisome proliferator-activated receptor alpha; SIRT1: sirtuin 1; PGC1α: proliferator-activated receptor gamma coactivator 1-alpha.

The HFD group significantly decreased the expression of adipocyte *PPARα*, *CPT1α, CPT2*, *SIRT1*, and *PGC1α* genes involved in fatty acid oxidation (FAO) compared to the ND group (Figure 4). The supplementation of AL significantly decreased *FAS* gene expression involved in lipogenesis, while increased FAO-associated gene expressions (*CPT1α* and *CPT2*) compared to the HFD. In addition, supplementation of AL-LS and AL-GJ significantly down-regulated *FAS* gene expression compared to that of LS and GJ. Especially, AL-GJ significantly increased *PPARα*, *CPT1α*, *CPT2,* and *PGC1α* gene expressions compared to the HFD group. AL-LS also markedly increased *CPT1α* expression compared with the HFD group. Furthermore, supplementation of AL-LSGJ significantly increased the *PPARα*, *CPT1α*, *CPT2*, and *SIRT1* gene expression compared to HFD, in addition to which the *SIRT1* gene expression was also significantly increased in AL-LSGJ compared to that in LS and AL.

## 4. Discussion

Probiotics and prebiotics have been consumed for centuries, either as natural components of food, or as fermented foods [19]. Combinations of probiotics and prebiotics are defined as synbiotics, which are able to enhance health and reduce disease related issues [20]. Synbiotics are being targeted more and more for their potential as functional food ingredients [21]. Previous studies have indicated that allulose may have beneficial effects on obesity related metabolic disturbances [11]. However, only a few studies have been conducted on the effects of allulose as prebiotics. Additionally, the previous studies have reported that *Lactobacillus sakei* LS03 and/or *Leuconostoc kimchi* GJ2 isolated from a traditional Korean fermented vegetable kimchi may improve adiposity, inflammation, and cardiovascular diseases [7,8,9]. Therefore, we investigated the levels of plasma, hepatic lipids, enzyme activities, and gene expressions to investigate the suitability of allulose as prebiotics for two probiotics *Lactobacillus sakei* LS303 and *Leuconostoc kimchi* GJ2. Results indicated that BW gain was significantly decreased in the AL group compared to the HFD group, it was tended to be lower in the AL-LS and AL-GJ groups than in the AL group, and the addition of allulose to two species of probioticss represented by AL-LSGJ group, led to a significant decrease in BW as well as BW gain compared to supplementation of AL alone. Similarly, the weight of total WAT was significantly lower in the AL-GJ group than in the GJ and AL groups. Supplementations of AL-GJ and AL-LSGJ significantly decreased the weight of total WAT, and AL-LSGJ markedly decreased not only total WAT but also visceral WAT weights compared to supplementation of AL alone. Adipose tissue, which is the major site of lipid storage, produces and releases a variety of pro-inflammatory and anti-inflammatory factors, including the adipokine leptin, adiponectin, resistin, as well as cytokines, such as IL-1β, IL-6, and TNF-α, among others [22,23]. In those with obesity, pro-inflammatory factors are released excessively and these excess amounts are considered to play an important role in the pathogenesis of obesity-related complications such as insulin resistance, type II diabetes, nonalcoholic fatty liver disease, and dyslipidemia [22]. During the development of obesity, plasma leptin levels are increased, while plasma adiponectin levels are decreased [24,25]. In this study, addition of allulose to HFD along with oral probiotics (AL-LS, AL-GJ, AL-LSGJ), as well as the allulose supplement alone, consistently resulted in the decrease of body weight, FER, body fat mass, as well as the concentration of plasma leptin, resistin and IL-1β. Leptin and IL-1β levels were significantly decreased by the addition of probiotic feeding of GJ to AL. Although the changes in plasma adiponectin levels were not dramatic, the ratio of leptin to adiponectin was lowered in the AL-, AL-LS-, AL-LS-, and AL-LSGJ groups. Among these 4 groups, the AL-LSGJ group which consisted of allulose and 2 species of probiotics were most effective to reduce some abnormalities of obesity. This observation suggests that allulose was effective in improving body weight and adiposity, revealed a synergy effect with probiotics, and synbiotic containing both allulose and 2 species of probiotics were most effective. Also, decreased adiposity by these synbiotics is partially linked to improvement of adipokines/cytokines dysregulation involved in inflammation.

Metabolic and endocrine functions of adipose tissues, which contribute to obesity related metabolic disorders, are partly associated with adipose tissue gene expression [26]. mRNA expression of *FAS* in epididymal white adipose tissue was related to adipogenesis. *FAS*, is one of the major genes related to fatty acid synthesis [27]. On the contrary, PPARα induces FAO by activating FAO-related genes, such as *CPT1α, CPT2*, and others [28,29]. *PGC1α* and *SIRT1* are important genes that induce transcription of mitochondrial FAO [30]. Interestingly, the supplementation of AL significantly down-regulated adipocyte *FAS* gene expression, while up-regulated adipocyte *CPT1α,* and *CPT2* expressions when compared to the HFD, thereby reducing adiposity. Moreover, the mRNA expression of *PGC1α* was significantly increased by the addition of allulose to the GJ treated mice (AL-GJ), and mRNA expression of *SIRT1* in the allulose supplemented with two probiotic species group (AL-LSGJ) was significantly up-regulated compared to the allulose group. The results of the present work suggest that allulose was effective to reduce body weight and adiposity by decreasing adipocyte lipogenesis and increasing FAO, and allulose exerted synergic effect with probiotics in the prevention of adiposity by further increasing the adipocyte gene expressions of *PGC1α* by AL-GJ, and *SIRT1* by AL-LSGJ.

Triglyceride, cholesterol, and other lipids are transported in the blood by lipoproteins, such as very low-density lipoprotein (VLDL), low-density lipoprotein (LDL) and HDL [31]. ApoA-1 is the principal apolipoprotein component of HDL-C, and ApoB-100 is a major apolipoprotein component of VLDL, intermediate-density lipoprotein (IDL), and LDL [32]. In general, obesity causes abnormalities in lipoprotein metabolism such as dyslipidemia and hyperlipidemia by increasing the synthesis of VLDL, LDL, ApoB, and total body cholesterol [33]. In the present study, supplementation of allulose and/or probiotics led to elevated HTR while significantly decreasing AI to levels when compared to HFD group. Supplementation of AL-GJ significantly decreased plasma TC, while significantly increased ApoA-I/ApoB ratio compared to that in the GJ and AL groups. Moreover, supplementation of AL-LSGJ dramatically decreased plasma TC and FFA levels, while markedly increasing the plasma Apo A-I level and ApoA-I/ApoB ratio compared to that in the LS, GJ and AL groups. It appears that when allulose was present as a prebiotic in the HFD with LS and GJ mixture, the overall effect was more beneficial than LS or GJ alone. In addition, supplementation of allulose to GJ-treated mice (AL-GJ) resulted in a decrease in plasma TC and ApoB, and an increase in ApoA-I and the ApoA-I/ApoB ratio, with the decreased total WAT weight, compared to the AL-LS group. Therefore, GJ probiotic appeared to be more responsive to the prebiotic allulose.

Liver plays an important role in lipid metabolism via pathways such as hepatic fatty acid synthesis and oxidation. However, obesity may lead to hepatic steatosis due to the accumulation of lipid droplets in the hepatocytes [34]. Both hepatic TG and FFA were significantly decreased by AL compared to the HFD. LS and GJ probiotics also significantly reduced hepatic TG and FFA compared to the HFD. In particular, hepatic TG, cholesterol, and FFA in the AL-LSGJ group were significantly decreased compared to the HFD group, and hepatic FFA in the AL-LSGJ group was also markedly decreased compared to the LS and AL groups. Such results could be partly due to alteration of the activities of hepatic lipid-regulating enzymes or the expression of hepatic genes. Based on G6PD and ME activities, fatty acid synthesis were significantly lowered by the AL supplement alone, while ME activity was significantly lowered by LS and GJ supplements, compared to the HFD group. In addition, combination of AL-GJ and AL-LSGJ led to a decrease in ME activity compared to that in AL and significantly recued G6PD activity compared to the LS and GJ groups. On the other hand, the hepatic enzyme activities, β-oxidation, related to fatty acid oxidation was tended to be higher in alluloase-supplemented groups (AL, AL-LS, AL-GJ, and AL-LSGJ) compared to HFD group. Also, supplementation of allulose to the GJ-treatment led to elevated CPT activity compared to that in mice fed HFD-based diet.

On the other hand, the hepatic enzyme activities, β-oxidation, and CPT, related to fatty acid oxidation were significantly increased by the of allulose supplementation to GJ-treated mice. Also, CPT activity was most significantly increased in the AL-GJ group and the mRNA expression of *CPT1α* and *CPT2* were significantly increased by supplementation of allulose to GJ-treated mice. Consequently, our study indicates that the most beneficial effects reaped by mice given the allulose supplement with both GJ and LG probiotics were the improvements seen in dyslipidemia and hepatic steatosis due to the inhibition of fat accumulation in the liver. Accordingly, although treatment with the probiotics GJ or LG may improve some obesity related metabolic markers, supplementation of allulose may further improve obesity related markers, thus supporting its role as a beneficial prebiotic. Based on the overall result, a combination of both probiotics and allulose may, in the least, produce a synergistic regulatory effect on body fat metabolism.

## 5. Conclusions

Results of the current study indicate that allulose, known as a functional sugar substitute with 0.2 kcal/g energy, exhibited a beneficial effect by lowering the body weight and abdominal fat mass in DIO mice. Furthermore, the anti-obesity effect of allulose was superior to that of probiotics (LS, GJ), and allulose had a synergy effect with probiotics in improving the deleterious effect of diet-induced obesity and its complications as evidenced by further decreased adiposity, and the levels of plasma lipid and pro-inflammatory adipokines/cytokines, and hepatic lipids by AL-GJ and/or AL-LSGJ when compared to the probiotics or allulose alone. When compared to AL, the supplementation AL-GJ significantly decreasing the total WAT weight by increasing adipocyte *PGC1α* mRNA expression, which contributed to decreased plasma TC, and increased ApoA-I/ApoB ratio. Especially, synbiotic containing both allulose and two species of probiotics (AL-LSGJ) was the most effective in the prevention of hepatic steatosis, adiposity and dyslipidemia. AL-LSGJ improved adiposity by increasing adipocyte *SIRT1* mRNA expression involved in FAO, which contributed to reduce the dyslipidemia and inflammation. Moreover, AL-LSGJ improved hepatic steatosis via decreased hepatic lipogenesis by suppressing the ME enzyme activity, which also contributed to improve dyslipidemia. These observations have suggested that allulose may act as a prebiotic for the two probiotics tested in the study. This new synbiotic mixture with allulose may help ameliorate the deleterious effects of diet-induced obesity and contribute to the growth of the food industry.

## Figures and Tables

**Table 1:** Composition of experimental diets with or without allulose supplement.

	ND	HFD	AL
Casein	20	20	20
D, L-Methionine	0.3	0.3	0.3
Corn starch	15	11.1	11.1
Sucrose	50	37	34
Cellulose	5	5	5
Corn oil	5	3	3
Lard		17	17
Mineral mix ^1^	3.5	4.2	4.2
Vitamin mix ^2^	1	1.2	1.2
Choline bitartrate	0.2	0.2	0.2
Cholesterol		1	1
Tert-Butylhydroquinone	0.001	0.004	0.004
Allulose			3
Total (%)	100	100	100

^1^ AIN-76 mineral mixture (Harlan Teklad Co., Madison, WI, USA). ^2^ AIN-76 vitamin mixture (Harlan Teklad Co., Madison, WI, USA). ND: normal diet; HFD: high-fat diet; AL: allulose.

**Table 2:** Primer sequences used for RT-qPCR validation of the microarray data.

Gene	Primer Direction	Primer Sequence
GAPDH	Forward	5′-AGGTCGGTGTGAACGGATTTG-3′
	Reverse	5′-TGTAGACCATGTAGTTGAGGTCA-3′
FAS	Forward	5′-GCTGCGGAAACTTCAGGAAAT-3′
	Reverse	5′-AGAGACGTGTCACTCCTGGACTT-3′
ACC1	Forward	5′-GCCTCTTCCTGACAAACGAG-3′
	Reverse	5′-TGACTGCCGAAACATCTCTG-3′
CPT1α	Forward	5′-ATCTGGATGGCTATGGTCAAGGTC-3′
	Reverse	5′-GTGCTGTCATGCGTTGGAAGTC-3′
CPT2	Forward	5′-GCCTGCTGTTGCGTGACTG-3′
	Reverse	5′-TGGTGGGTACGATGCTGTGC-3′
PPARα	Forward	5′-GGCACCCTCACATCATCAAACTG-3′
	Reverse	5′-TGGAACAGACGGCGGCTTTC-3′
SIRT1	Forward	5′-TGTGAAGTTACTGCAGGAGTGTAAA-3′
	Reverse	5′-GCATAGATACCGTCTCTTGATCTGAA-3′
PGC1α	Forward	5′-AAGTGTGGAACTCTCTGGAACTG-3′
	Reverse	5′-GGGTTATCTTGGTTGGCTTTATG-3′

GAPDH: glyceraldehyde-3-phosphate dehydrogenase; FAS: fatty acid synthase; ACC1: acetyl-CoA; carboxylase 1; CPT1α and CPT2: carnitine palmitoyltransferase 1-alpha and 2; PPARα: peroxisome proliferator-activated receptor alpha; SIRT1: sirtuin 1; PGC1α: peroxisome proliferator-activated receptor gamma coactivator 1-alpha.

**Table 3:** Effect of synbiotic supplementation for 12 weeks on organ and white adipose tissue weights.

	ND	HFD	LS	GJ	AL	AL-LS	AL-GJ	AL-LSGJ
**Liver**	3.27 ± 0.05 ***	4.67 ± 0.20	4.18 ± 0.16	4.27 ± 0.25	4.56 ± 0.12	4.49 ± 0.10	4.31 ± 0.19	4.18 ± 0.11
**Kidney**	0.92 ± 0.02 ***	0.72 ± 0.01 ^a^	0.76 ± 0.03 ^ab^	0.76 ± 0.03 ^ab^	0.83 ± 0.03 ^bc^	0.85 ± 0.02 ^c^	0.90 ± 0.03 ^c^	0.89 ± 0.05 ^c^
**Epididymal WAT**	3.11 ± 0.21 ***	6.05 ± 0.11 ^c^	6.03 ± 0.27 ^c^	5.98 ± 0.12 ^bc^	5.89 ± 0.25 ^bc^	5.43 ± 0.18 ^abc^	5.18 ± 0.34 ^ab^	4.84 ± 0.25 ^a^
**Perirenal WAT**	0.40 ± 0.03 ***	0.94 ± 0.04 ^d^	0.79 ± 0.07 ^bcd^	0.90 ± 0.11 ^cd^	0.71 ± 0.06 ^abc^	0.66 ± 0.05 ^ab^	0.57 ± 0.07 ^ab^	0.54 ± 0.04 ^a^
**Mesenteric WAT**	0.88 ± 0.07 ***	1.97 ± 0.12 ^e^	1.77 ± 0.19 ^de^	1.60 ± 0.14 ^cd^	1.26 ± 0.13 ^bc^	1.19 ± 0.08 ^abc^	1.07 ± 0.10 ^ab^	0.86 ± 0.06 ^a^
**Subcutaneous WAT**	2.67 ± 0.24 ***	6.10 ± 0.22 ^b^	5.59 ± 0.38 ^b^	5.65 ± 0.32 ^b^	5.68 ± 0.50 ^b^	5.17 ± 0.27^ab^	4.37 ± 0.41 ^a^	4.35 ± 0.31 ^a^
**Retroperitoneal WAT**	0.75 ± 0.06 ***	1.44 ± 0.03 ^b^	1.34 ± 0.05 ^ab^	1.28 ± 0.11 ^ab^	1.38 ± 0.07 ^ab^	1.30 ± 0.06 ^ab^	1.19 ± 0.09 ^a^	1.21 ± 0.08 ^a^
**Interscapular WAT**	1.53 ± 0.13 ***	3.27 ± 0.08 ^c^	2.77 ± 0.20 ^b^	2.70 ± 0.13 ^b^	2.57 ± 0.24 ^b^	2.33 ± 0.14 ^ab^	2.02 ± 0.19 ^a^	2.03 ± 0.16 ^a^
**Total WAT**	9.34 ± 0.68 ***	19.63 ± 0.40 ^d^	19.29 ± 0.65 ^d^	18.11 ± 0.64 ^cd^	17.49 ± 1.14 ^cd^	16.49 ± 0.44 ^bc^	14.69 ± 1.00 ^ab^	14.09 ± 0.63 ^a^
**Visceral WAT**	5.14 ± 0.35 ***	10.39 ± 0.18 ^d^	9.93 ± 0.51 ^cd^	9.76 ± 0.22 ^cd^	9.23 ± 0.43 ^bc^	8.58 ± 0.32 ^ab^	8.01 ± 0.56 ^ab^	7.45 ± 0.36 ^a^

Data are mean ± standard error of the mean (S.E.); Significant differences ND versus HFD are indicated; *** *p* < 0.001; ^abcde^ Means with different superscript letters are significantly different among the groups (*p* < 0.05); ND: normal diet (5% fat, *w*/*w*); HFD: high fat diet (20% fat, 1% cholesterol, *w*/*w*); LS: HFD + *Lactobacillus sakei* LS03 10^9^ cfu/day; GJ: HFD + *Leuconostoc kimchi* GJ2 10^9^ cfu/day; AL: HFD + 3% allulose; AL-LS: AL + *Lactobacillus sakei* LS03 10^9^ cfu/day; AL-GJ: AL + *Leuconostoc kimchi* GJ2 10^9^ cfu/day; AL-LSGJ: AL + *Lactobacillus sakei* LS03 + *L. Leuconostoc kimchi* GJ2 10^9^ cfu/each/day; FER, food efficiency ratio = body weight gain/energy intake per day; WAT: white adipose tissue.

**Table 4:** Effect of synbiotic supplementation for 12 weeks on plasma lipid profiles in diet-induced obese mice.

	ND	HFD	LS	GJ	AL	AL-LS	AL-GJ	AL-LSGJ
**TG (mmol/L)**	0.80 ± 0.05 **	0.98 ± 0.05 ^b^	0.87 ± 0.05 ^ab^	0.83 ± 0.05 ^a^	0.78 ± 0.05 ^a^	0.75 ± 0.06 ^a^	0.82 ± 0.03 ^a^	0.73 ± 0.03 ^a^
**Total-C (mmol/L)**	3.37 ± 0.14 ***	5.29 ± 0.22 ^c^	4.62 ± 0.26 ^bc^	4.56 ± 0.18 ^bc^	4.81 ± 0.29 ^bc^	4.85 ± 0.26 ^bc^	4.32 ± 0.30 ^a^	4.32 ± 0.15 ^a^
**FFA (mmol/L)**	0.71 ± 0.01 *	0.77 ± 0.02 ^b^	0.74 ± 0.02 ^ab^	0.72 ± 0.02 ^ab^	0.73 ± 0.02 ^ab^	0.71 ± 0.02 ^ab^	0.75 ± 0.02 ^ab^	0.69 ± 0.02 ^a^
**HDL-C (mmol/L)**	1.03 ± 0.06 ***	1.62 ± 0.04 ^a^	1.64 ± 0.11 ^a^	1.76 ± 0.07 ^ab^	1.75 ± 0.09 ^ab^	1.71 ± 0.06 ^ab^	1.58 ± 0.08 ^a^	1.88 ± 0.06 ^b^
**nonHDL-C (mmol/L)**	2.45 ± 0.12 ***	3.79 ± 0.19 ^b^	2.96 ± 0.17 ^a^	2.78 ± 0.16 ^a^	3.10 ± 0.21 ^a^	3.15 ± 0.14 ^a^	2.59 ± 0.20 ^a^	2.69 ± 0.13 ^a^
**HTR (%)**	30.63 ± 0.63	29.29 ± 0.73 ^a^	35.46 ± 0.64 ^b^	36.80 ± 1.17 ^b^	35.80 ± 1.15 ^b^	35.04 ± 1.51 ^b^	37.03 ± 1.03 ^b^	37.90 ± 1.07 ^b^
**AI**	2.24 ± 0.07	2.46 ± 0.10 ^b^	1.80 ± 0.04 ^a^	1.69 ± 0.10 ^a^	1.87 ± 0.09 ^a^	1.82 ± 0.11 ^a^	1.76 ± 0.08 ^a^	1.72 ± 0.07 ^a^
**ApoA-I (mg/dL)**	44.72 ± 0.43	44.87 ± 0.42 ^b^	43.39 ± 0.28 ^a^	44.51 ± 0.32 ^ab^	44.61 ± 0.75 ^ab^	43.91 ± 0.51 ^ab^	45.20 ± 0.42 ^b^	49.05 ± 0.60 ^c^
**ApoB 100 (mg/dL)**	2.74 ± 0.24 ***	5.91 ± 0.61 ^b^	5.10 ± 0.38 ^ab^	4.62 ± 0.82 ^ab^	5.10 ± 0.25 ^ab^	5.07 ± 0.59 ^ab^	3.78 ± 0.35 ^a^	3.37 ± 0.37 ^a^
**ApoA-I/ApoB**	19.01 ± 0.97 ***	8.56 ± 0.72 ^a^	8.85 ± 0.51 ^a^	11.97 ± 1.43 ^ab^	8.89 ± 0.39 ^a^	10.13 ± 1.43 ^ab^	13.15 ± 1.47 ^bc^	16.30 ± 2.09 ^c^

Data are mean ± S.E.; Significant differences ND versus HFD are indicated; * *p* < 0.05, ** *p* < 0.01, *** *p* < 0.001; ^abc^ Means with different superscript letters are significantly different among the groups (*p* < 0.05); ND: normal diet (5% fat, *w*/*w*); HFD: high fat diet (20% fat, 1% cholesterol, *w*/*w*); LS: HFD + *Lactobacillus sakei* LS03 10^9^ cfu/day; GJ: HFD + *Leuconostoc kimchi* GJ2 10^9^ cfu/day; AL: HFD + 3% allulose; AL-LS: AL + *Lactobacillus sakei* LS03 10^9^ cfu/day; AL-GJ: AL + *Leuconostoc kimchi* GJ2 10^9^ cfu/day; AL-LSGJ: AL + *Lactobacillus sakei* LS03 + *L. Leuconostoc kimchi* GJ2 10^9^ cfu/each/day; HTR: (HDL-C /Total-C) ×100; AI: atherogenic index = (Total-C) − (HDL-C)/(HDL-C); ApoA-I: ApoB ratio; TG: triglyceride; FFA: free fatty acid; Total-C: total-cholesterol; HDL-C: high-density lipoprotein cholesterol.
